# Study on mechanism of low bioavailability of black tea theaflavins by using Caco-2 cell monolayer

**DOI:** 10.1080/10717544.2021.1949074

**Published:** 2021-08-31

**Authors:** Fengfeng Qu, Zeyi Ai, Shuyuan Liu, Haojie Zhang, Yuqiong Chen, Yaomin Wang, Dejiang Ni

**Affiliations:** a Key Laboratory of Horticultural Plant Biology, College of Horticulture and Forestry Sciences, Ministry of Education, Huazhong Agricultural University, Wuhan, China; b College of Horticulture, Qingdao Agricultural University, Qingdao, China

**Keywords:** Caco-2 cells, absorption, transport, theaflavins, metabolites

## Abstract

This study aimed to clarify the bioavailability mechanism of theaflavins by using the Caco-2 monolayer *in vitro* model. Prior to the transport of theaflavin (TF), theaflavin-3-gallate (TF3G), theaflavin-3’-gallate (TF3’G), and theaflavin-3, 3’-digallate (TFDG), we found the cytotoxicity of theaflavins was in the order of TF3’G > TFDG > TF3G > TF, suggesting the galloyl moiety enhances the cytotoxicity of theaflavins. Meantime, the galloyl moiety made theaflavins unstable, with the stability in the order of TF > TFDG > TF3G/TF3’G. Four theaflavins showed poor bioavailability with the *P*_app_ values ranging from 0.44 × 10^−7^ to 3.64 × 10^−7^ cm/s in the absorptive transport. All the theaflavins showed an efflux ratio of over 1.24. And it is further confirmed that P-glycoprotein (P-gp), multidrug resistance associated proteins (MRPs) and breast cancer resistance protein (BCRP) were all shown to contribute to the efflux transport of four theaflavins, with P-gp playing the most important role, followed by MRPs and BCRP. Moreover, theaflavins increased the expression of P-gp, MRP1, MPR3, and BCRP while decreased the expression of MRP2 at the transcription and translation levels. Additionally, the gallated theaflavins were degraded into simple theaflavins and gallic acids when transported through Caco-2 monolayers. Overall, the structural instability, efflux transporters, and cell metabolism were all responsible for the low bioavailability of four theaflavins in Caco-2 monolayers.

## 1. Introduction

As a fully fermented tea, black tea is characterized with red liquor, red leaves, and sweet taste, and the fermentation process is crucial for the formation of its unique flavor and health effect due to the generation of theaflavins, thearubigins, and theabrownins. Theaflavins, the oxidation products of a pair of epimerized catechins, are responsible for the golden color and astringent taste of black tea (Wan, 2003). Furthermore, theaflavins, especially the gallated theaflavins, were found to be positively correlated with the bioactivities of black tea (Qu et al., 2020). In black tea, theaflavins are primarily composed of four monomers: theaflavin (TF), theaflavin-3-gallate (TF3G), theaflavin-3′-gallate (TF3’G), and theaflavin-3, 3′-digallate (TFDG), which vary in the position and number of galloyl moiety. The hydroxyl groups attached to the theaflavin skeleton and galloyl moiety enable theaflavins to effectively scavenge free radicals and promote the various bioactivities of theaflavins (Xie et al., 2018). For instance, theaflavins can suppress the adhesion and invasion of hepatoma cells by scavenging hydroxyl radicals and reactive oxygen species (ROS) (Zhang et al., 2000). Additionally, theaflavins can trigger a reduction of ROS in human colon adenocarcinoma cancer cells due to their excellent radical-scavenging ability (Tan et al., 2019). Furthermore, theaflavins are reported to develop resistance against bacteria, virus, inflammation, hyperglycemia, and cardiovascular disease (Liu et al., 2013). As one of the major naturally functional components in black tea, theaflavins have a promising prospect of clinical application.

It is well acknowledged that the key to the unique effects of bioactive components is maintaining sufficient concentration and long residence time at their action sites. Bioavailability is associated with the concentration of the given components or their related metabolites absorbed in the target organ (García-Arieta, 2014). Previous reports have shown that theaflavins have poor systematic bioavailability. It is reported that only a small amount of theaflavins can be detected in the plasma and urine samples of healthy volunteers after 2 hours of consumption of 700 mg mixed theaflavins (Mulder et al., 2001). The amount of TFDG in tissue samples collected from mice treated with decaffeinated black tea (50 mg/g diet) for two weeks was less than 1 nmol/g tissue (Henning et al., 2006). Many factors have been found to affect the final bioavailability of phenolic compounds, such as food matrix, biological transporters, molecular structures, metabolic enzymes, and intestinal microbiota (Ferreira et al., 2017). However, the mechanism of poor bioavailability of theaflavins in human, especially in the intestine, the major absorption site for xenobiotics, remains unclear.

Studies have shown that for small organic molecules, the monolayer permeability of human colon adenocarcinoma cell line (Caco-2) has an excellent correlation with the permeability of the rat intestinal perfusion system and *in vivo* human absorption (Stewart et al., 1995; Yee, 1997). Since this discovery, Caco-2 monolayers have been widely used to predict the oral absorption of bioactive compounds. In the present study, a Caco-2 monolayer model was established to figure out the mechanism of the reported low absorption of theaflavins in human intestine, which would provide a theoretical basis for the application of theaflavins in pharmaceutical and health care products.

## 2. Materials and methods

### 2.1. Materials

TF, TF3G, TF3’G, and TFDG were purchased from Shanghai Yuanye Biological Technology Company Limited (Shanghai, China). Caco-2 cells were purchased from cell bank of Chinese Academy of Science, Shanghai. Dulbecco modified eagle medium (DEME), fetal bovine serum, Hank’s balanced salts solution (HBSS) and other cell culture medium components were all purchased from Hyclone (LA, USA). Verapamil (Vep) and MK-571 were obtained from Sigma-Aldrich (DA, Germany). Cyclosporin A (CsA) was obtained from Cayman Chemical (MI, USA). Fumitremorgin C (FTC) was obtained from MedcChemExpress (NJ, USA). The primary antibodies of multidrug resistance asscioated proteins (MRP1, 14685S; MRP2, 12559S; MRP3, 14182S), P-glycoprotein (P-gp, 13342S), and breast cancer resistance protein (BCRP, 42078S) were purchased from Cell Signaling Technology (MA, USA). Horseradish peroxidase (HRP)-conjugated goat anti-rabbit (SA00001-2) was purchased from Proteintech (Wuhan, China). Radio immunoprecipitation assay (RIPA) lysis buffer and phenylmethanesulfonyl fluoride (PMSF) were purchased from Beyotime (Shanghai, China). Poly-vinylidene fluoride (PVDF) membranes, enhanced chemiluminescence reagents (ECL) and other western reagents were obtained from Service Biological Technology Co., Ltd (Wuhan, China). BCA protein assay kit and cell counting kit-8 (CCK-8) were purchased from Nanjing Jiancheng Bioengineering Institute (Nanjing, China). Cell RNA extraction kit, cDNA synthesis kit and SYBR Green qPCR Mix were purchased from Aidlab (Beijing, China). All the primers were synthesized by Sangon Biotech (Shanghai, China).

### 2.2. Cell culture

Human Caco-2 cells were well cultured in complete DMEM media, supplemented with 10% fetal bovine serum, 5% nonessential amino acids, 5% L-glutamine, 5% penicillin and streptomycin, and 0.2% plasmocin prophylactic (2.5 mg/mL). Caco-2 cells were grown in 25 cm^2^ Corning flasks (NY, USA) in a CO_2_ incubator (MCO-15AC, Sanyo, Osaka, Japan) at 37 °C under 5% humidified CO_2_. The cells were routinely sub-cultured at 70–80% confluence. All experiments were performed using cells from passages 40 to 55.

### 2.3. Cytotoxicity study

Standard stock solutions (1000 μM) of each theaflavin were prepared in 0.1% dimethyl sulfoxide (DMSO) in deionized water, and diluted with fresh DMEM to 100, 200, 300, 400, and 500 µmol/L respectively. Each inhibitor was prepared as instructions. The cytotoxicity of theaflavins and four inhibitors of multidrug resistant associated proteins to Caco-2 cells was examined by CCK-8 method (Song et al., 2014). Briefly, Caco-2 cells were inoculated onto 96-well microtiter plates at a density of 1 × 10^4^ cells per well. After incubation at 37 °C for 24 h, cells were incubated for another 24 h with different concentrations of theaflavins, followed by adding 10 μL CCK-8 solution and 1 h incubation at 37 °C. The absorbance (OD) value was determined at 450 nm using a microplate reader (ELX-800, Bio-Tek Instruments Inc., Winooski, USA). Four inhibitors were used in this study: Vep, inhibitor of P-gp; CsA, inhibitor and substrate of P-gp; MK-571, inhibitor of MRP1, MRP2 and MRP3; FTC, inhibitor of BCRP (Silbermann et al., 1989; König et al., 1999; Renes et al., 1999; Ali et al., 2017). The cytotoxicity of Vep, CsA, MK-571 or FTC was also determined by CCK-8 assay. Figure S2 showed the effective concentration of Vep, CsA, MK-571, and FTC was 100, 100, 100, and 10 μM respectively. To determine the effects of inhibitors on the cytotoxicity of theaflavins, cells were incubated with both different theaflavin monomers (100, 200, 300, 400, and 500 μM) and different inhibitors. IC_50_, the half inhibitory concentration, was calculated from the cell viability. The cell viability was calculated by the following equation:

(1) $$\text{Cell viability }\left(\%\right)=\left({\text{OD}}_{\text{sample}}-{\text{ OD}}_{\text{blank}}\right)/\left({\text{OD}}_{\text{control}}-{\text{ OD}}_{\text{blank}}\right)\times \text{1}00\%$$

where OD_sample_ is the absorbance value of cell + sample + complete medium; OD_blank_, the absorbance value of complete medium; OD_control,_ the absorbance value of cell + complete medium.

### 2.4. Preparation of test solutions and evaluation of stability

Standard stock solutions of each theaflavin were diluted with HBSS and DMEM respectively, obtaining final test solutions of 200 μM. The recovery rate of theaflavins in HBSS and in DMEM, in the absence of cells, was analyzed by HPLC after incubation at 37 °C for 0.5, 1, 2, 3, 4, 8, 12, and 24 hours, respectively. The recovery rate was used to measure the stability and calculated as follows:

(2) $$\text{Recovery rate}={\text{C}}_{\text{t}}/{\text{C}}_{0}{}_{}\times \text{ 1}00\%$$

where C_t_ is the final concentration at different incubation time (μM); C_0_, the initial concentration (μM).

### 2.5. Transepithelial transport of theaflavins in Caco-2 cells

For transport studies, Caco-2 cells were seeded at a density of 3 × 10^5^ cells per well in Transwell^®^ inserts (polycarbonate membrane, 24 mm i.d., 0.4 μm pore size, Corning Costar Corp., NY, USA) in 6-well plates (Zhang et al., 2004). After differentiation over a period of 21–23 days post-confluency, Figure S1 indicated that our cell monolayers showed good integrality with the transepithelial electrical resistance values exceeding 500 Ω cm^2^ and the alkaline phosphatase activity of apical (AP) side to basolateral (BL) side more than 5.5 (Zhu & Yang, 2012). The inserts were washed twice with HBSS buffer and equilibrated at 37 °C for 30 min. Standard stock solutions of each theaflavin were diluted to 200 μM with HBSS. For absorptive transport, theaflavins in 1.5 mL HBSS was added into the AP side and 2.6 mL HBSS was added into the BL side of all inserts. For secretory transport, 1.5 mL HBSS and 2.6 mL theaflavins in HBSS were added into AP side and BL side, respectively. Samples were collected from the receiving chamber after 0.5, 1, 2, 3, or 4 h incubation and then were lyophilized at −110 °C in a CoolSafe freeze dryer (LaboGene ApS, Bjarkesvej, Denmark) for HPLC analysis. The recovery rate of theaflavins in the transport was calculated from the above Equation (2).

**Figure 1.**
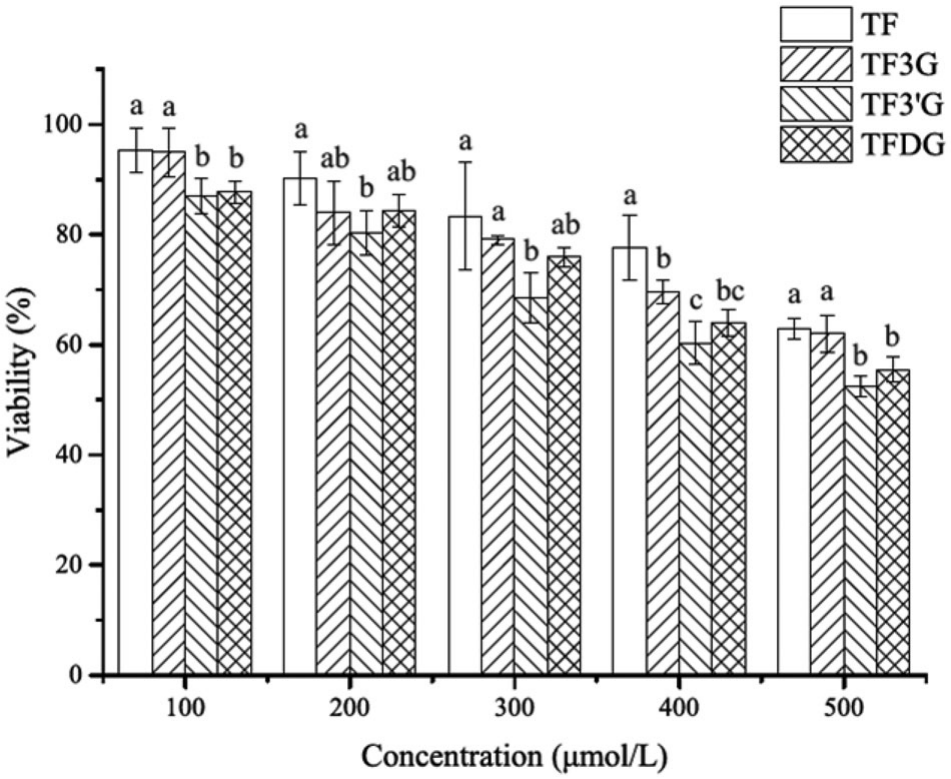
Effects of TF, TF3G, TF3’G, and TFDG on viabilities of Caco-2 cells. Results are expressed as means ± SD (*N* = 3). Different small letters indicate significant difference between different treatments at *p*<.05.

Prior to transport inhibition experiment, cells were preincubated for 30 min with 100 μM Vep, 100 μM CsA, 100 μM MK-571, or 10 μM FTC in both sides (Vaidyanathan & Walle, 2001; Wang et al., 2008). Then, the absorptive and secretory experiments were performed in the presence of inhibitors as described above. Samples were collected from the receiving chamber after 2 h incubation and then were lyophilized at −110 °C for HPLC analysis.

The apparent permeability coefficient (*P*_app_, cm/s) was calculated as follows:

(3) $$P_{\text{app}}=\text{ dQ}/\text{dt}\times \text{V}/(\text{A}\times {\text{C}}_{0})$$

where dQ/dt is the variation in concentration on the receiving side over time (μM/s); V, the volume of the solution collected from the receiving chamber (cm^3^); A, the area of the membrane (4.67 cm^2^); C_0,_ the initial concentration of theaflavins in the donor chamber (μM).

The efflux ratio was calculated as follows:

(4) $$\text{Efflux ratio}={\text{ P}}_{\text{app}(\text{BL}-\text{AP})}/{\text{P}}_{\text{app}(\text{AP}-\text{BL})}$$

where P_app_, apparent permeability coefficient; AP-BL, apical to basolateral transport; BL-AP, basolateral to apical transport.

### 2.6. Intracellular accumulation of theaflavin monomers

Caco-2 cells were seeded at a density of 8 × 10^4^ cells per well in 6-well plates (Ni et al., 2020). After differentiation over a period of 21 days, cells were incubated with 2.0 mL of 200 μM theaflavins at 37 °C. After 2 h incubation, phosphate buffer was added to wash cells in the plate. Then, cell lysates were prepared with RIPA lysis buffer containing 1 mM PMSF as a protease inhibitor and centrifugated at 12,000 rpm for 30 min. Total protein contents were quantified using a BCA estimation kit according to the manufacturer’s instructions (Beyotime). Theaflavins were quantified by HPLC analysis and the intracellular accumulation was expressed as µg theaflavin/mg protein.

### 2.7. HPLC analysis

Theaflavins were characterized by high performance liquid chromatography (HPLC, Agilent 1100VL, Agilent Technologies Inc., CA, USA). Briefly, samples were extracted with 70% methanol, centrifuged at 685 × g for 10 min, and filtered through a 0.45 μm membrane (Nylon 66). The separation was performed by an ODS reversed-phase column (4.6 mm i.d.×250 mm, SB-C18, Agilent Technologies Inc., CA, USA) at a flow rate of 0.7 mL/min. Chromatographic conditions were slightly modified according to China National Standard (GB/T 30483-2013, GAQSIQ, P. R. China, 2013). Samples were eluted with a gradient of solvent A (100 mL water with 9 mL acetonitrile and 2 mL acetic acid) and B (100 mL acetonitrile with 17.8 mL water and 2 mL acetic acid) as follows: 0–5 min, 95% A; 5–10 min, 90% A; 10–20 min, 70% A; 20–25 min, 65% A; 25–30 min, 70% A; 30–35 min, 100% A. Theaflavins were detected using an ultraviolet detector at a wavelength of 278 nm.

### 2.8. Western blot

Caco-2 cells were seeded at a density of 8 × 10^4^ per well in 6-well plates, followed by 21 days of culture and then 2 hours of treatment at 37 °C with or without different theaflavin monomers (200 μM). Cell lysates were prepared with RIPA lysis buffer. Total protein contents were quantified using a BCA estimation kit according to the manufacturer’s instructions. Cell protein (50 μg) was loaded onto SDS polyacrylamide gel (5% stacking gel; 8% separating gel) and separated by electrophoresis for 1.5 h at 20 mA. Next, the separated proteins were transferred onto the PVDF membrane in ice bath for 1.5 h at 120 mA, followed by blocking the membrane with 5% nonfat milk in Tris-buffered saline containing 0.1% Tween-20 (TBST) for 1 h at room temperature. After blocking, the blots were incubated overnight at 4 °C with the primary antibody of P-gp, MRP1, MRP2, MRP3, BCRP (1:200) or β-actin (1:40,000), followed by washing with TBST and incubation for 1 h at room temperature with HRP-conjugated goat anti-rabbit (1:3,000). Immunoreactive proteins were visualized by the ECL detection system, band intensity was quantified by Image Lab (Bio-Rad, CA, USA), and the protein contents were normalized to β-actin.

### 2.9. Quantitative real-time PCR

Cell culture and treatment were performed as described above in 2.6. Total RNA was extracted from Caco-2 cells by the cell RNA extraction kit, and then reverse-transcribed by the cDNA synthesis kit. Gene amplification and quantitative detection were performed as previously described (Ai et al., 2019). All the sequences of primers used in this study are shown in Table S3. Target mRNA levels were normalized to β-actin mRNA levels. Data were calculated by the 2^−ΔΔCT^ method.

### 2.10. HPLC-MS/MS analysis

TF3G, TF3’G and TFDG were dissolved in DMSO (≤0.5%), and then, diluted with HBSS. Caco-2 cells were incubated with 200 µM TF3G, TF3’G and TFDG at 37 °C for 2 h. Samples collected from both AP side and BL side were measured by HPLC with MS detection, and the metabolites were determined in terms of the standard TF, TF3G, TF3’G, TFDG, and gallic acid (Table S2). Briefly, each sample (3 μL) was separated by HPLC- MS/MS (6420 A, Agilent Technologies Inc., CA, USA) with a poroshell column (2.1 mm i.d.×150 mm, 2.7 μm, SB-C18, Agilent Technologies Inc., CA, USA) at a flow rate of 0.3 mL/min. The samples were eluted with a gradient of solvent A (100 mL water with 0.1 mL acetic acid) and B (100 mL acetonitrile) as follows: 0–10 min, 80% A; 10–13 min, 50% A; 13–20 min, 80% A. The MS parameters for electrospray ionization (ESI in positive mode) were set as follows: curtain gas 15 psi, ion spray voltage +4000 V in positive mode and −3500 V in negative mode, ESI capillary 3.5 kV, gas temperature 350 °C, source temperature 300 °C, and nebulization gas 65 psi.

### 2.11. Statistical analysis

Data were expressed as the mean ± standard deviation (SD). Results were analyzed using a one-way analysis of variance (ANOVA) followed by Fisher’s least significant difference (LSD) procedure. Differences were considered significant at *p*<.05. Statistical analysis was performed using SPSS statistical software (SPSS, Chicago, IL, USA). All experiments were carried out three times.

## 3. Results

### 3.1. The cytotoxicity of theaflavins

Theaflavins have been reported to inhibit the proliferation of cancer cells by regulating the growth factor receptor, interfering in the ubiquitin-proteasome pathway, reducing energy supply, blocking cell cycle, and inducing cell apoptosis (Li et al., 2015). In order to minimize the influence of cytotoxicity of theaflavins on their transport manner in Caco-2 cells, the safe transport concentration of theaflavins was determined. In Figure 1, the cytotoxicity of theaflavins was seen to be in the order of TF3’G > TFDG > TF3G > TF, suggesting the cytotoxicity of theaflavins could be enhanced by the galloyl moiety and TF3’G was a more potential inhibitor against proliferation of Caco-2 cells than other theaflavin monomers. Furthermore, Figure 1 also revealed that the viability of Caco-2 cells was more than 80% with the treatment of theaflavins at 200 µmol/L. Thus, we consider 200 µmol/L to be a safe effective concentration in the transport assay.

### 3.2. The stability of theaflavins

**Figure 2.**
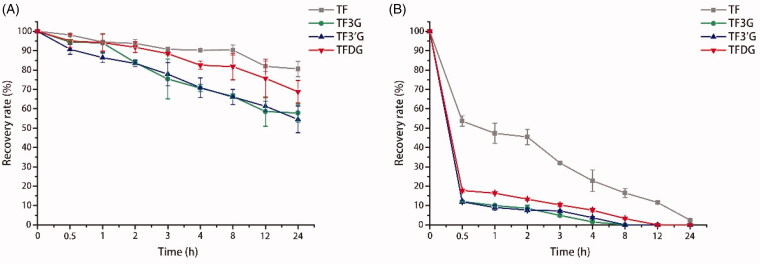
Recovery rate of TF, TF3G, TF3’G, and TFDG in different medium. (A) Recovery rate of theaflavins in HBSS; (B) Recovery rate of theaflavins in DMEM. Results are expressed as means ± SD (*N* = 3).

The structural stability of active substances is one of the factors affecting their bioavailability. To choose a suitable dissolving medium, the stability of four theaflavin monomers was analyzed by determining their recovery rate in pH 6.0 HBSS and pH 6.0 DMEM without cells. Four theaflavin monomers showed a significant decrease in stability with increasing incubation time, which followed the order of TF > TFDG > TF3G/TF3’G (Figure 2), indicating a higher structural stability of non-gallated theaflavin than gallated theaflavin. After 2 h incubation, all theaflavin monomers showed excellent stability with a recovery rate of over 80% in HBSS (Figure 2(A)), while theaflavins were extremely unstable in DMEM, with a recovery rate of less than 15% for TF3G, TF3’G, and TFDG and 45% for TF (Figure 2(B)). At the end of 24 h incubation, the recovery rate of the four theaflavin monomers was still much higher in HBSS than in DMEM, indicating HBSS is a better choice than DMEM for dissolving theaflavins.

### 3.3. Theaflavins transport

**Figure 3.**
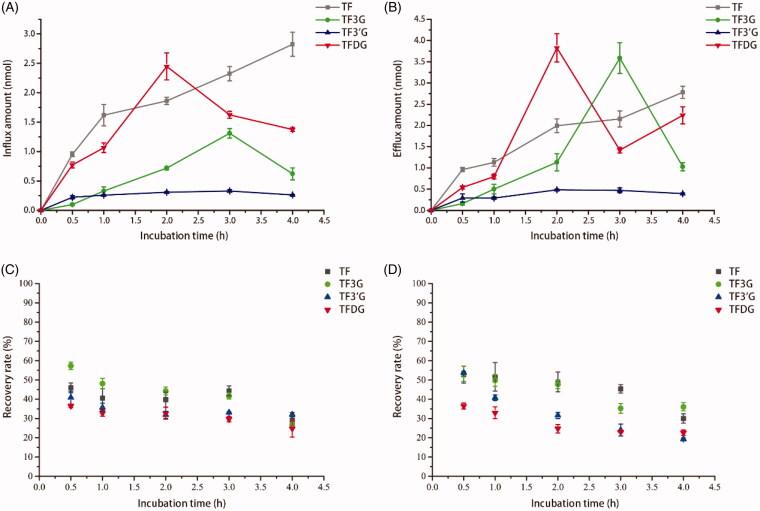
Transepithelial transport and recovery rate of TF, TF3G, TF3’G and TFDG in Caco-2 cells. **(**A) Influx amounts of theaflavins in apical to basolateral transport; (B) Efflux amounts of theaflavins in basolateral to apical transport; (C) Recovery rate of theaflavins in apical to basolateral transport; (D) Recovery rate of theaflavins in basolateral to apical transport. Results are expressed as means ± SD (*N* = 3).

In this study, the characteristic of theaflavin transport under different incubation time was investigated. As shown in Figures 3(A,B), both the influx and efflux amount of TF increased in a time-dependent manner, while the bi-directional transport amount of TF3G increased firstly and then decreased with increasing incubation time, peaking at 3 h with 1.30 ± 0.08 nmol for absorptive transport and 3.59 ± 0.36 nmol for secretory transport. TFDG showed a similar transport manner as TF3G, with both the absorption peak (2.44 ± 0.23 nmol) and the secretion peak (3.83 ± 0.33 nmol) present at 2 h. Meanwhile, TF3’G showed no great variations in the amount of both the absorptive or secretory transport relative to other theaflavins. It has been proved that the *P*_app_ values of active substances are well correlated with their bioavailability. Generally, the *P*_app_ values below 1 × 10^−6^ cm/s means extremely poor bioavailability of active substances (Yee, 1997). In Table 1, the *P*_app_ values of the four theaflavin monomers were seen to follow the order of TFDG > TF > TF3G > TF3’G and range from 0.44 × 10^−7^ to 3.64 × 10^−7^ cm/s in the absorptive transport, suggesting the extremely low bioavailability of theaflavins. The mono-gallated theaflavins, especially TF3’G, were shown to be poorly absorbed and utilized.

**Table 1.** Effects of multidrug resistance associated protein inhibitors on the transport of theaflavins across Caco-2 cell monolayers after 2 h incubation.

Inhibitor	TF			TF3G			TF3’G			TFDG		
	*P*_app (AP−BL)_	*P*_app (BL−AP)_	Efflux ratio	*P*_app (AP−BL)_	*P*_app (BL−AP)_	Efflux ratio	*P*_app (AP−BL)_	*P*_app (BL−AP)_	Efflux ratio	*P*_app (AP−BL)_	*P*_app (BL−AP)_	Efflux ratio
Ctrl	2.77 ± .09b	3.42 ± .14b	1.24 ± .09b	1.07 ± .04c	1.68 ± .30c	1.58 ± .30a	0.44 ± .03d	0.72 ± .03d	1.62 ± .15a	3.64 ± .34a	5.69 ± .49a	1.57 ± .08a
Vep	4.15 ± .14**	3.01 ± .12*	0.73 ± .05**	2.03 ± .08**	1.73 ± .31	0.86 ± .16*	0.55 ± .03*	0.59 ± .02**	1.08 ± .10**	3.57 ± .33	5.46 ± .47	1.53 ± .08
MK-571	4.07 ± .14**	3.04 ± .13*	0.59 ± .19**	1.15 ± .05	2.41 ± .43	2.09 ± .4	0.51 ± .03*	0.67 ± .02	1.33 ± .12	3.89 ± .36	5.24 ± .46	1.35 ± .07*
CsA	5.65 ± .19**	3.48 ± .14	0.62 ± .05**	1.30 ± .05**	1.46 ± .26	1.13 ± .22	0.54 ± .03*	0.84 ± .03**	1.55 ± .14	4.00 ± .37	6.32 ± .55	1.58 ± .09
FTC	3.10 ± .10*	3.24 ± .13	1.05 ± .08	1.09 ± .04	1.53 ± .27	1.41 ± .27	0.54 ± 0.03*	0.67 ± .02	1.26 ± .11*	3.75 ± .35	5.52 ± .48	1.47 ± .08

*Note*: Ctrl: theaflavins with no inhibitor; Vep: Vep + theaflavins; CsA: CsA + theaflavins; FTC: FTC + theaflavins; *P*_app (AP-BL)_ values (×10^−7 ^cm/s) represent the *P*_app_ values in the absorptive transport; *P*_app (BL-AP)_ values (×10^−7 ^cm/s) represent the *P*_app_ values in the secretory transport; *p < .05 and **p < .01 indicate significant differences (inhibitor group) relative to the control group; different lowercase letters indicate significant difference at *p* < .05 among different theaflavin monomers.

Also, we analyzed the recovery of theaflavins in the transport of Caco-2 cells. As shown in Figure 3, the recovery of theaflavins gradually decreased with the incubation time increased from 0.5 to 4 h. And after 2 h incubation, the recovery of theaflavins was 39.8% for TF, 44.2% for TF3G, 31.8% for TF3’G, 32.7% for TFDG in absorptive transport (Figure 3(C)) and 48.9% for TF, 47.6% for TF3G, 31.6% for TF3’G, 24.7% for TFDG in secretory transport (Figure 3(D)). Additionally, we determined the intracellular accumulation of four theaflavin monomers, and only a minute amount of TF3G was detected inside Caco-2 cells (Table S1). Inferred form Figure 2(A), 9.3% of TF, 24.6% of TF3G, 22.1% of TF3’G, and 11.6% of TFDG was degraded due to the structural instability. The recovery of theaflavins in the transport should be 100% according to the law of mass conservation. However, the sum of all the recovery of theaflavins was considerably lower than 100%, indicating a portion of theaflavins are unaccounted for in the transport across Caco-2 cell monolayers.

### 3.4. Inhibition of theaflavins transport

ATP binding cassette (ABC) superfamily transporters, including P-glycoprotein (ABCB subfamily), multidrug resistance associated protein (ABCC subfamily) and breast cancer resistance protein (ABCG subfamily), are a large group of membrane proteins, which can pump a wide spectrum of substrates and xenobiotics out of Caco-2 cells (Chan et al., 2004). In our study, the effects of P-glycoprotein (P-gp), multidrug resistance associated proteins (MRP1, MRP2, MRP3) and breast cancer resistance protein (BCRP) on the transport of theaflavins were further investigated by using Vep, CsA, MK-571 and FTC. In Figure S2, the four inhibitors of efflux transporters were shown to have low toxicity to Caco-2 cells at a given concentration. Table 2 showed Vep, MK-571, CsA, and FTC significantly decreased the IC_50_ values of theaflavins in Caco-2 cells, suggesting the involvement of efflux transporters in reducing the cytotoxicity of theaflavins.

**Table 2.** Effects of four multidrug resistance associated protein inhibitors on the cytotoxicity of theaflavins.

Inhibitors	TF IC_50_ (µmol/L)	TF3G IC_50_ (µmol/L)	TF3’G IC_50_ (µmol/L)	TFDG IC_50_ (µmol/L)
Ctrl	681.41 ± 19.25a	640.96 ± 7.80a	521.33 ± 31.95ab	579.57 ± 34.73b
VEP	517.37 ± 66.25bc	453.73 ± 13.21b	466.87 ± 53.26bc	508.76 ± 40.64b
MK-571	462.66 ± 80.79c	585.09 ± 78.70a	423.85 ± 34.98c	373.09 ± 38.84c
CsA	583.97 ± 26.85b	446.11 ± 30.10b	574.96 ± 84.28a	702.16 ± 69.41a
FTC	492.35 ± 34.21bc	507.88 ± 15.91b	432.00 ± 30.69bc	580.45 ± 83.91b

*Note*: Ctrl: theaflavins with no inhibitor; Vep: Vep + theaflavins; CsA: CsA + theaflavins; FTC: FTC + theaflavins; different lowercase letters in the same column indicates significant difference at *p*<.05.

The effects of multidrug resistance protein inhibitors on the transport of theaflavins across Caco-2 cell monolayers are shown in Table 1. Results further confirmed the involvement of the efflux transporters in the transport of theaflavins. In terms of absorptive transport, verapamil showed a 1.50-, 1.90-, and 1.24-fold increase in the influx of TF, TF3G and TF3’G, respectively; MK-571 showed the strongest promoting effect on the influx of TF, with a 1.47-fold increase; CsA was considered as a potential promoter for improving the bioavailability of all theaflavins except TFDG and increased the influx of TF by 2.0 times; FTC seemed to only increase the influx of TF and TF3’G. Our data suggest that P-gp, MRP1, MRP2, MRP3, and BCRP were all positively related to the excretion of theaflavins, and P-gp played the most important role, followed by MRPs and BCRP. Moreover, co-incubation with inhibitors significantly reduced the secretory transport of theaflavins, leading to an obvious decrease in the efflux ratio of theaflavins. Taken together, TF, TF3G, TF3’G and TFDG can all be secreted by efflux transporters when transport across Caco-2 monolayers, leading to their poor absorption and utilization.

### 3.5. Effects of theaflavins on the expression of MRP1, MRP2, MRP3, P-gp, and BCRP

**Figure 4.**
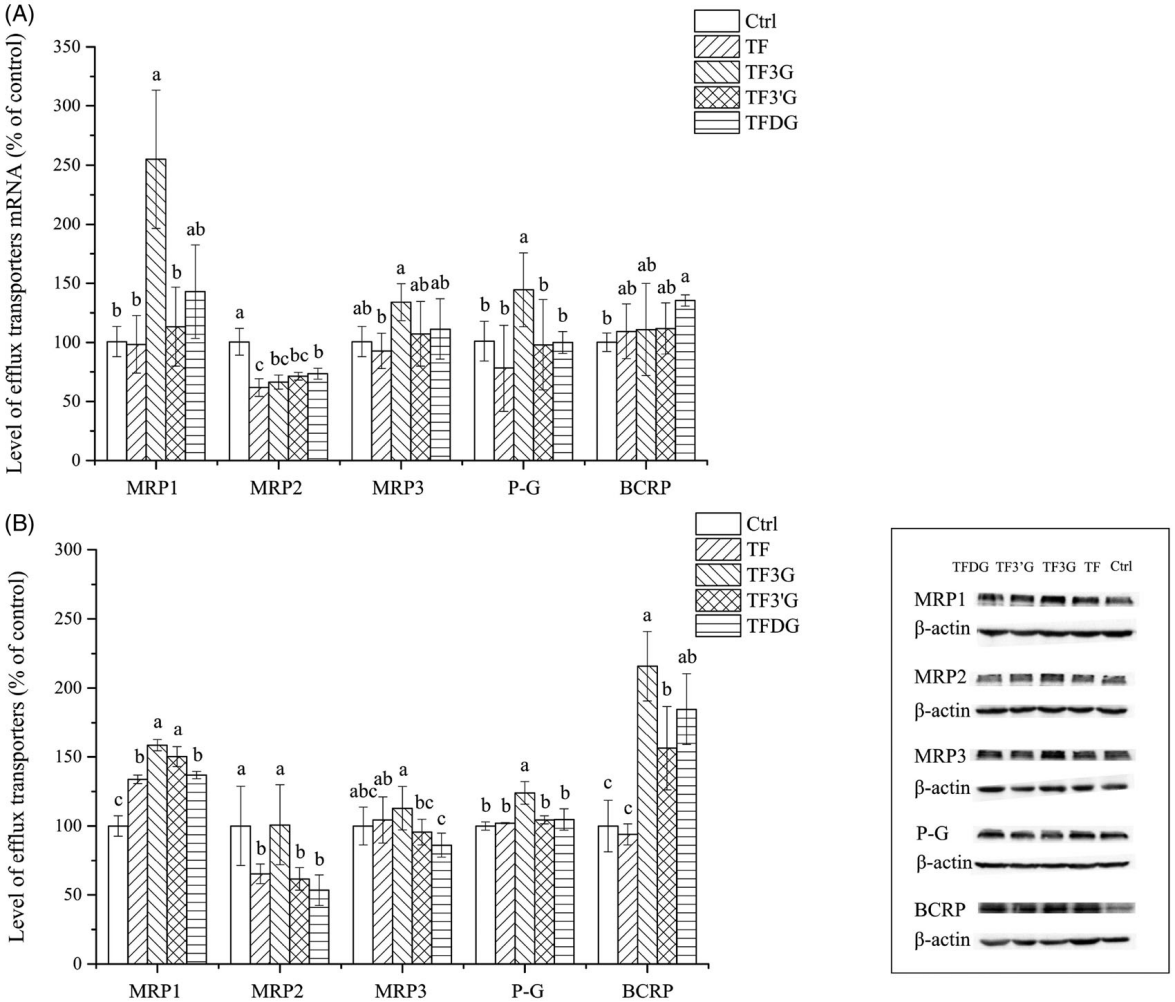
Effects of different theaflavin monomers on the mRNA and protein levels of MRP1, MRP2, MRP3, P-gp and BCRP in Caco-2 cells. (A) Effect of theaflavins on mRNA levels of efflux transporters. (B) Effect of theaflavins on protein levels of efflux transporters. Ctrl: Caco-2 cells were incubated with HBSS; TF, TF3G, TF3’G and TFDG: Caco-2 cells were incubated with TF, TF3G, TF3’G and TFDG. Results are expressed as means ± SD (*N* = 3). Different small letters indicate significant difference between different treatments at *p*<.05.

The effects of theaflavins on the expression of major ABC transporters in Caco-2 cells were explored by measuring the mRNA and protein levels of MRP1, MRP2, MRP3, P-gp, and BCRP. In Figure 4(A), TF3G was seen to increase the mRNA levels of MRP1, MRP3, and P-gp by 154.97%, 34.01%, and 44.45%, respectively, and TFDG upregulated the mRNA level of BCRP by 35.49%. However, the mRNA levels of MRP2 were down-regulated by the four theaflavin monomers. Particularly, under TF treatment, the transcriptional level of MRP2 in Caco-2 cells was decreased by 38.73%. The effects of different theaflavin monomers on the translational levels of efflux transporters are shown in Figure 4(B). TF3G increased the protein levels of MRP1, P-gp and BCRP by 58.36%, 23.94%, and 115.78%, respectively, and TF3’G and TFDG also increased the protein levels of MRP1 and BCRP. However, all the theaflavins except for TF3G markedly decreased the protein expression of MRP2. Generally, albeit to a small degree, theaflavins, especially TF3G, can up-regulate the expression of MRP1, MRP3, P-gp, and BCRP, while down-regulate the expression of MRP2 at the transcription and translation levels. It is inferred that the theaflavins are not just the substrates but also the regulatory factors for efflux transporters.

### 3.6. Metabolites of theaflavins

**Figure 5.**
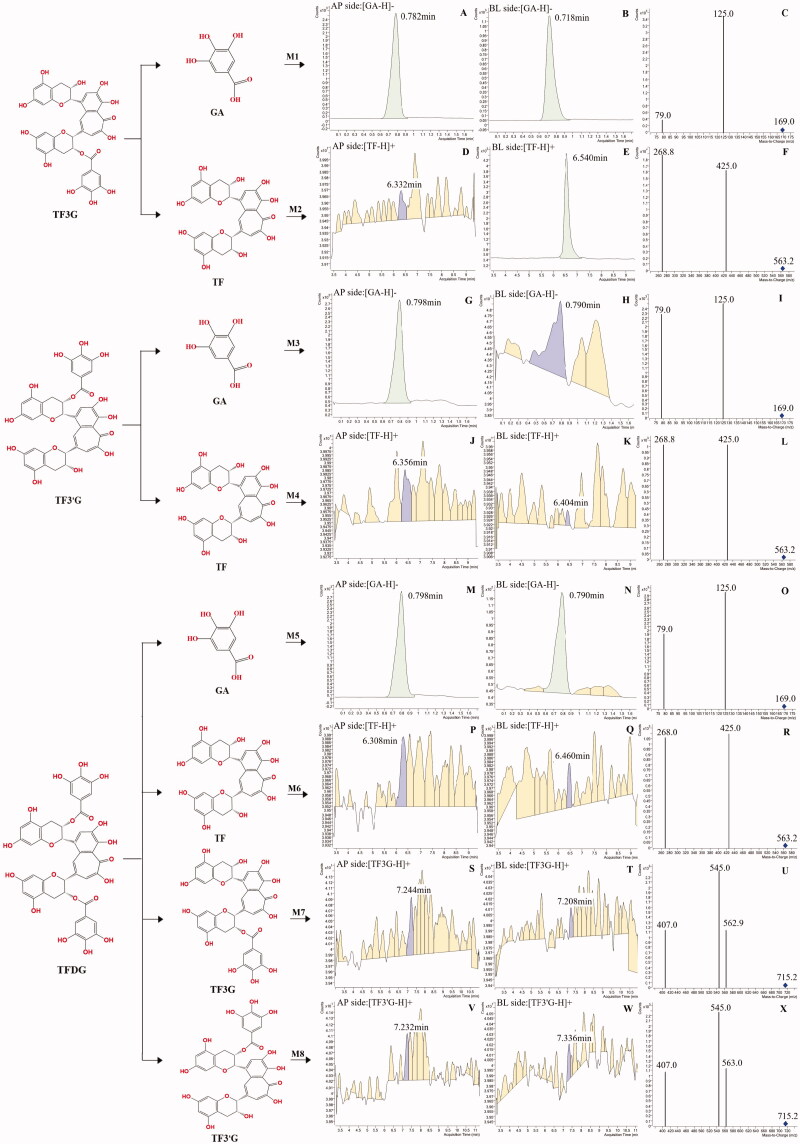
Identification of gallated theaflavins metabolites in the absorptive transport of Caco-2 monolayers. A, D, G, J, M, P, S, V, the chromatograms of gallated theaflavins metabolites in the apical side; B, E, H, K, N, Q, T, W, the chromatograms of gallated theaflavins metabolites in the basolateral side; C, F, I, L, O, R, U, X, the fragmentation patterns of gallated theaflavins metabolite.

The metabolites of theaflavins in the absorptive transport of Caco-2 monolayers after 2 h were identified in our study. In TF3G-treated samples, two new peaks, M1 (*m/z* 169.0, RT 0.782 min, Figure 5(A,B)) and M2 (*m/z* 563.2, RT 6.332 min, Figure 5(D,E)), were found in both the AP side and BL side in the Caco-2 monolayer. The retention time (RT) and MS^2^ spectra of peaks M1 and M2 were consistent with those of authentic gallic acid (GA) and TF in Table S2, respectively, confirming that TF3G could be metabolized to GA and TF by Caco-2 cells and transported from AP side to BL side. Likewise, TF3’G could be bio-transformed to GA (peak M3, Figure 5(G,H)) and TF (peak M4, Figure 5(J,K)) and transported from AP side to BL side. In Figure 5(M–Q), the peaks M5 and M6 observed in the TFDG-treated samples were also identified as GA and TF, respectively. In the TFDG-treated samples, the two new peaks, M7 (*m/z* 715.2, RT 7.244 min, Figure 5(S,T)) and M8 (*m/z* 715.2, RT 7.232 min, Figures 5(V,W)), showed almost the same retention time and MS^2^ spectra of TF3G and TF3’G, respectively, implying that GA, TF, TF3G, and TF3’G are the major metabolites of TFDG in Caco-2 cells. Our results suggest that the galloyl-theaflavins can be degraded into simple theaflavins and gallic acids when transported across Caco-2 monolayers, leading to a low bioavailability of galloyl-theaflavins. However, the metabolites of TF cannot be recognized in this study and need further structural analysis.

## 4. Discussion

In this study, we tried to figure out the reason for the low bioavailability of theaflavins by using Caco-2 cells. Initially, we found that theaflavins could inhibit the proliferation of Caco-2 cells, which was consistent with previous studies reporting that theaflavins have inhibitory effect on the proliferation of human colon cancer cell HT-29 and COLO205 (Hiroshige et al., 2003; Friedman et al., 2007). Furthermore, TF3’G, TFDG and TF3G showed stronger cytotoxicity than TF, suggesting the cytotoxicity of theaflavins could be enhanced by the galloyl moiety. This result agreed well with the finding of Tan et al (2019), who reported the inhibitory effect of theaflavins on the proliferation of human colon adenocarcinoma cancer SW480 cells was in the order of TF3’G > TFDG > TF3G > TF. The inhibition of antioxidants against the proliferation of cancer cells are based on their ability in scavenging free radicals (Valko et al., 2007). Theaflavins are reported to downregulate the expression of cell cycle-related proteins by inhibiting ROS formation, thus inducing G0/G1-phase cell cycle arrest (Tan et al., 2019). The radical scavenging ability of polyphenols is shown to depend on the position and quantity of hydroxyl groups in the aromatic ring (Tu et al., 2012). All these reports suggest that the hydroxyl groups in theaflavins may be responsible for the death of Caco-2 cells.

In alkaline conditions, theaflavins can be easily degraded to theanaphthoquinone due to one-electron oxidation reaction (Su et al., 2003; Jhoo et al., 2005). Thus the pH value of transport solution was adjusted to 6.0. And it is reported that, compared to gallated theaflavins, non-gallated theaflavins are more resistant to degradation (Su et al., 2003; Pereira-Caro et al., 2017), which was consistent with our results. Our data revealed that all the theaflavin monomers were more stable in HBSS than in DMEM. The instability of theaflavins in DMEM is mainly due to the essential nutrients for cells. For instance, theaflavins can be precipitated by amino acids and proteins, or complex with Cu^2+^ and Fe^3+^, thereby leading to a low recovery rate of theaflavins (Yoshinori et al., 1971; Miller et al., 1996). And the major complexing site of theaflavins with metal ions are reported to be the adjacent hydroxyl groups in the B ring of theaflavins (Brown et al., 1998).

To analyze the bioavailability of theaflavins, the transport manners of theaflavins were studied in our study. TF was transported in both dose- and time-dependent manner. The flux amounts of TF3G and TFDG increased first and then decreased with the increasing incubation time, and the decrease was likely due to the structural instability and cell metabolism. No significant variation was found in the transport of TF3’G. All the theaflavin monomers had extremely low bioavailability with the range of *P*_app_ values from 0.44 × 10^−7^ to 3.64 × 10^−7^ cm/s. Polyphenols can form hydrogen bond with the polar head groups of lipids at the cell membrane interface and induce changes in the membrane physical properties, thus preventing the access of exogenous molecules to the cell (Verstraeten et al., 2003). Previous study demonstrated that free hydroxyl groups decreased the bioavailability of anthocyanins (Yi et al., 2006). In our study, TF was higher than the gallated theaflavins in the absorptive amount, probably because less hydroxyl groups can promote the absorption of theaflavins. The efflux ratio values of the four theaflavins were shown to exceed 1.0, suggesting the dominance of efflux transport in the transport of theaflavins across Caco-2 monolayers. As previously reported, many efflux proteins, such as P-gp, MRPs and BCRP, are secreted by Caco-2 cells and they are proved to be a critical determinant of oral bioavailability (Chan et al., 2004). Catechins could be bio-transformed into methylated and sulfated catehins in the transported of Caco-2 cells (Zhang et al., 2004; Ai et al., 2019). Hence, we deduced that the extremely low absorption of theaflavins, was likely to be correlated with efflux transporters and cell metabolism.

To further investigate the reasons for the poor bioavailability of theaflavins in Caco-2 cells, the effects of P-gp, MRP1, MRP2, MRP3 and BCRP on the transport of theaflavins were explored in this study. And we found that P-gp, MRP1, MRP2, MRP3 and BCRP are associated with the reduced absorption of the four theaflavin monomers, with P-gp playing the most important part, followed by MRPs and BCRP. Studies have shown a stronger effect of P-gp, MRP2 and BCRP on pumping epicatechin than epicatechin gallate (Kadowaki et al., 2008; Ai et al., 2019). Epicatechin is not only a substrate of P-gp, but can also bind and activate its allosteric sites, thus enhancing the efflux activity of P-gp (Wang et al., 2002). Galloyl groups are considered as the major limiting factors to the secretory transport of gallated catechins (Kadowaki et al., 2008). In the present study, P-gp and MRPs exhibited the strongest secretory effect on TF, but the weakest effect on TFDG, possibly due to the inhibition of galloyl groups on the activity of efflux transporters. Interestingly, TF3’G was shown as the only substrate of BCRP. Studies have shown that Ko143 (a derivative of FTC) and anthocyanins bring about conformational changes of BCRP dimers, resulting in an increase of substrate influx by BCRP (Özvegy-Laczka et al., 2005; Dreiseitel et al., 2009). Hence, we speculated that the increase of the influx of theaflavins caused by FTC might be correlated to the conformational alterations of BCRP.

In the accumulation tests, the low amounts of cells might be a reason that no theaflavin monomer except TF3G was detected in cells. While, it can be more attributed to the barrier of cell membrane (Verstraeten et al., 2003; Yi et al., 2006), efflux function and metabolism of cells.

Furthermore, we found theaflavins were not only the substrates of efflux transporters, but could also increase the expression of MRP1, MRP3, P-gp and BCRP to accelerate the efflux of theaflavins. Similar results were also found in the interaction between catechins and P-gp, MRP1 and MRP2 (Wang et al., 2002; Jungil et al., 2003; Vaidyanathan & Walle, 2003; Ai et al., 2019). Studies have shown that the expression of efflux transporters can be regulated by many factors, such as cytokines, cell stress, and transcription factors (Kast et al., 2002; Wang et al., 2013; 2018). Aconitum alkaloids have been found to increase the expression of P-gp in Caco-2 cells *via* activating pregnane X receptor (PXR) and constitutive androstane receptor (CAR) (Wu et al., 2016). The expression of MRP2 is also regulated by PXR and CAR (Kast et al., 2002). Some transcription factors like aromatic hydrocarbon receptor (AHR) and peroxisome proliferative activated receptor (PPAR) can mediate the transcriptional activation of BCRP in intestinal cells (Hirai et al., 2007; Tan et al., 2010). We suspect that theaflavins, especially TF3G, up-regulate the expression of efflux transporters by activating the transcription factors mentioned above. Additionally, the expression of efflux transporters is related to proteasomal activity (Farabegoli et al., 2010). Epigallocatechin gallate (EGCG) exhibits strong inhibitory activity against a purified 20S and 26S proteasome in intact tumor cells, while decreasing the number of hydroxyl groups in B- or D-ring or modifying the ester bond of EGCG will result in a decrease in the proteasome inhibitory activity (Dou et al., 2008). It has been reported that the gallated theaflavins has greater potency to inhibit the activity of proteasome than non-gallated theaflavins, suggesting that the ester bonds and galloyl groups are vital to the proteasome-inhibitory activities of theaflavins (Lin et al., 2006; Mujtaba & Dou, 2012). And this might be one of the reasons for the higher expression of P-gp, MRP1 and BCRP in Caco-2 cells under the treatment of gallated theaflavins. The specific pathways by which theaflavins regulate the expression of efflux transporters remain an untapped area and more efforts should be made in the future.

As mentioned previously, theaflavins could be self-degraded due to the structural instability. But the recovery rate of theaflavins in the transport of Caco-2 monolayers was far lower than the degradation rate, indicating some theaflavins were missing. Previous studies showed the metabolic enzymes secreted by the highly differentiated Caco-2 cells was in generally comparable to those secreted by human intestinal tissue (Vaessen et al., 2017). As precursors of theaflavins, catechins can be sulfated and methylated by metabolic enzymes in the transport of Caco-2 monolayer (Ai et al., 2019). Based on the above considerations, the ‘disappeared’ theaflavins may be metabolized by Caco-2 cells throughout the transport process. And we confirmed that theaflavins could be bio-transformed by Caco-2 cells. We found the major metabolic manner of gallated theaflavins was degalloylation. Earlier studies have shown that TF, TF3G, TF3’G and GA are the major metabolic products of TFDG in mouse feces (Chen et al., 2011) which is consistent with our results. Sulfate conjugation and methylation are the major metabolic pathways for catechins in Caco-2 cells, indicating that phase II metabolism-related enzymes, such as glucuronosyltransferase, glutathione-S-transferase, and N-acetyltransferase are involved in the metabolism of catechins (Ai et al., 2019). However, in the present study, the conjugated metabolites like glucuronidated, sulfated and methylated metabolites of TF, TF3G, TF3’G or TFDG were not detected in either the AP side or the BL side in Caco-2 monolayers, suggesting that the theaflavins are not direct participants of phase II metabolism. Furthermore, intestinal microorganisms have been reported to play a vital role in improving the intestinal uptake of high-molecular-weight polyphenols (Lipinski et al., 2001; Selma et al., 2009). Non-gallated theaflavin is comparatively resistant to degradation by colonic bacteria with a 67% recovery after 24 h incubation, while most of the gallated theaflavins can be degraded by colonic bacteria to form gallic acids (Pereira-Caro et al., 2017). We conjectured that the ester bond between galloyl groups and C ring might be the major target site of cell metabolism.

**Figure 6.**
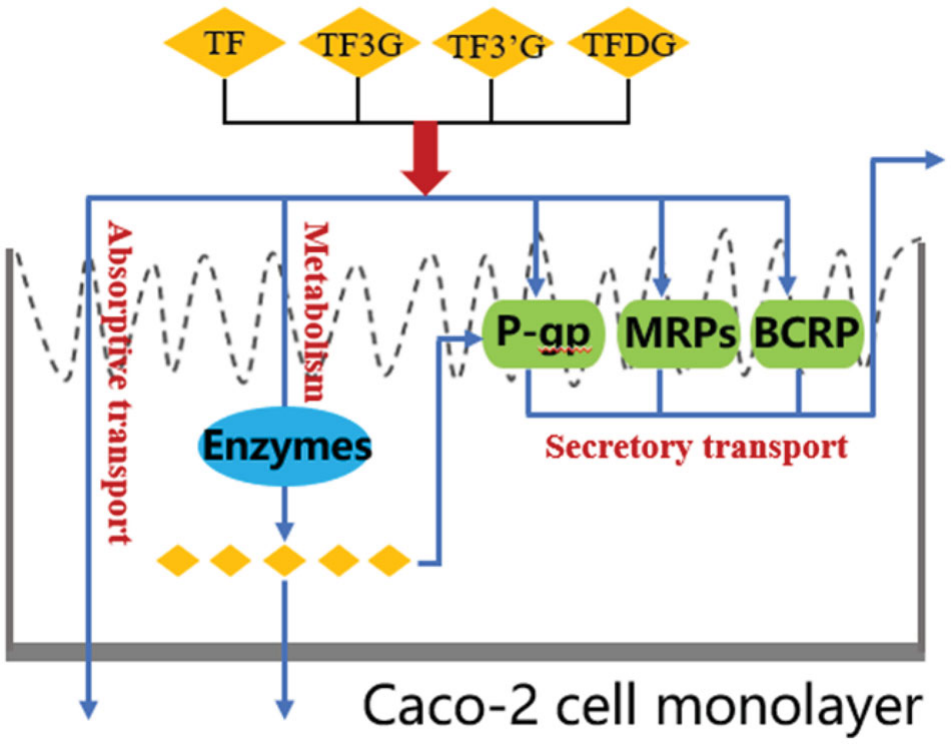
Transport route of theaflavins in Caco-2 cell monolayers.

In conclusion, the transport pathways of theaflavins across Caco-2 monolayers are shown as Figure 6: very few of theaflavins are absorbed; theaflavins are secreted by efflux transporters; theaflavins are metabolized by enzymes. And the efflux transporters and metabolic enzymes are mainly responsible for the low absorption of theaflavins when transported across Caco-2 monolayers.

## Supplementary Material

Supplemental Material

## Appendix

Contents of theaflavins absorbed in Caco-2 cells (µg/mg protein) (Table S1), transition ions and mass-spectrometry parameters of TF, TF3G, TF3’G, TFDG and gallic acid (Table S2), names and sequences of the primers used for quantitative real-time PCR (Table S3), determination of TEER values (A) and the ratio of the alkaline phosphatase activity from AP side to BL side (B) in Caco-2 cell monolayers at a different growth time (Figure S1), effects of four multidrug resistant associated protein inhibitors on the viabilities of Caco-2 cells. (A) verapamil; (B) MK-571; (C) CsA; (D) FTC (Figure S2).
