# Supplementary material for: Study on mechanism of low bioavailability of black tea theaflavins by using Caco-2 cell monolayer
Source: Drug Deliv. 2021 Aug 31;28(1):1737–47. doi: 10.1080/10717544.2021.1949074 (PMC8409943; doi:10.1080/10717544.2021.1949074)
Supplement: Supplemental Material [file IDRD_A_1949074_SM3381.docx]

Table S1 Contents of theaflavins absorbed in Caco-2 cells (µg/mg protein)

|  | TF | TF3G | TF3’G | TFDG |
| --- | --- | --- | --- | --- |
| Absorption | - | 1.675 ± 0.097 | - | - |

Note: “-” represents undetectable amount.

Table S2 Transition ions and mass-spectrometry parameters of TF, TF3G, TF3’G, TFDG and gallic acid. Bold font signifies the major MS/MS fragments.

| Compound | Retention time (min) | ESI mode | Detected mass (m/z) | MS^2^  (m/z) | Fragmentor (V) | Collision energy (eV) |
| --- | --- | --- | --- | --- | --- | --- |
| TF | 6.46 | + | 565.2 | **427**, 277, 139 | 120 | 14, 8, 36 |
| TF3G | 7.11 | + | 717.1 | **579**, 277, 139 | 140 | 12, 14, 36 |
| TF3’G | 7.38 | + | 717.2 | **579**, 277, 139 | 140 | 12, 12, 46 |
| TFDG | 6.01 | + | 869.2 | **743**, 277, 139 | 150 | 10, 14, 46 |
| Gallic acid | 0.79 | - | 169.0 | **125**, 79 | 80 | 12, 28 |

Table S3 Names and sequences of the primers used for quantitative real-time PCR

| Primers | Sequence (5’-3’) |
| --- | --- |
| MRP1/ABCC1 | F: 5’- GGGCTGCGGAAAGTCGT-3’ |
|  | R: 5’- AGCCCTTGATAGCCACGTG-3’ |
| MRP2/ABCC2 | F: 5’- TGAGCAAGTTTGAAACGCACAT-3’ |
|  | R: 5’- AGCTCTTCTCCTGCCGTCTCT-3’ |
| MRP3/ABCC3 | F: 5’-GTCCGCAGAATGGACTTGAT-3’ |
|  | R: 5’-TCACCACTTGGGGATCATTT-3’ |
| MDR1/ABCB1 | F: 5’-GCCAAAGCCAAAATATCAGAC-3’ |
|  | R: 5’-TTCCAATGTGTTCGGCAT -3’ |
| BCRP/ABCG2 | F: 5’-TGCAACATGTACTGGCGAAGA-3’ |
|  | R: 5’-TCTTCCACAAGCCCCAGG-3’ |
| β-Actin | F: 5’-AGCGAGCATCCCCCAAAGTT-3’  R: 5’-GGGCACGAAGGCTCATCATT-3’ |


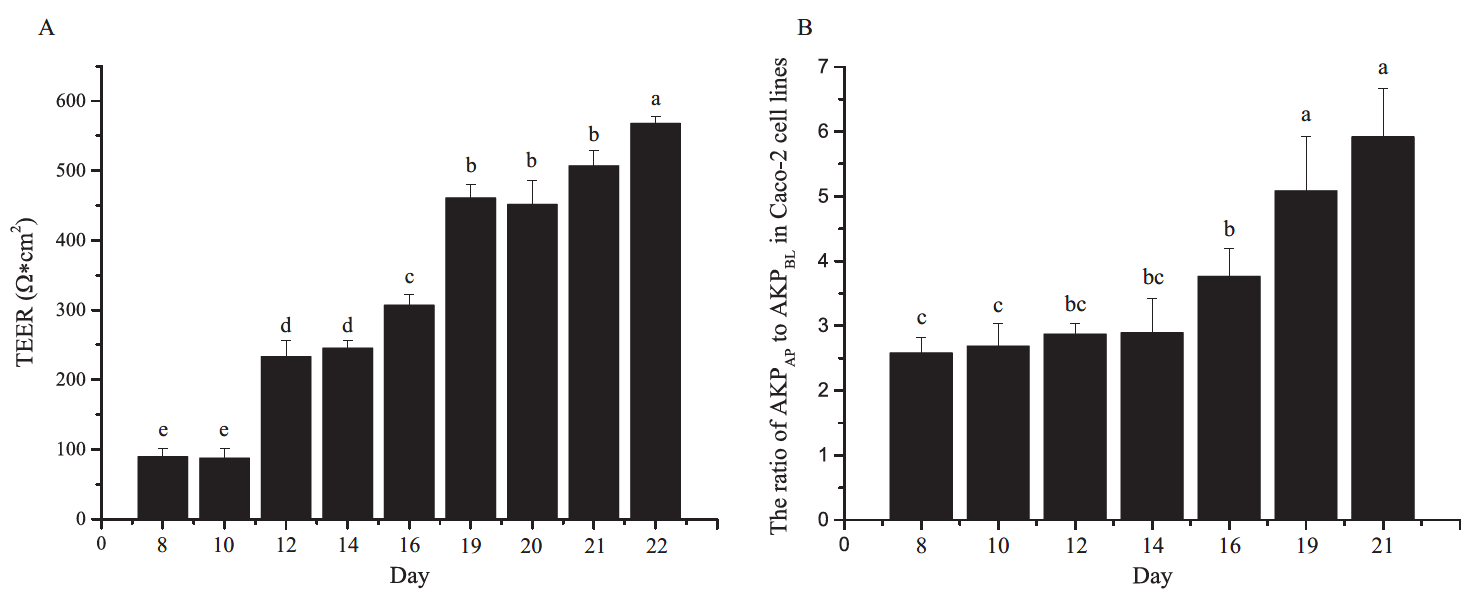
Fig. S1 Determination of TEER values (A) and the ratio of the alkaline phosphatase activity from AP side to BL side (B) in Caco-2 cell monolayers at a different growth time. Different small letters indicate significant difference between different treatments at p < 0.05


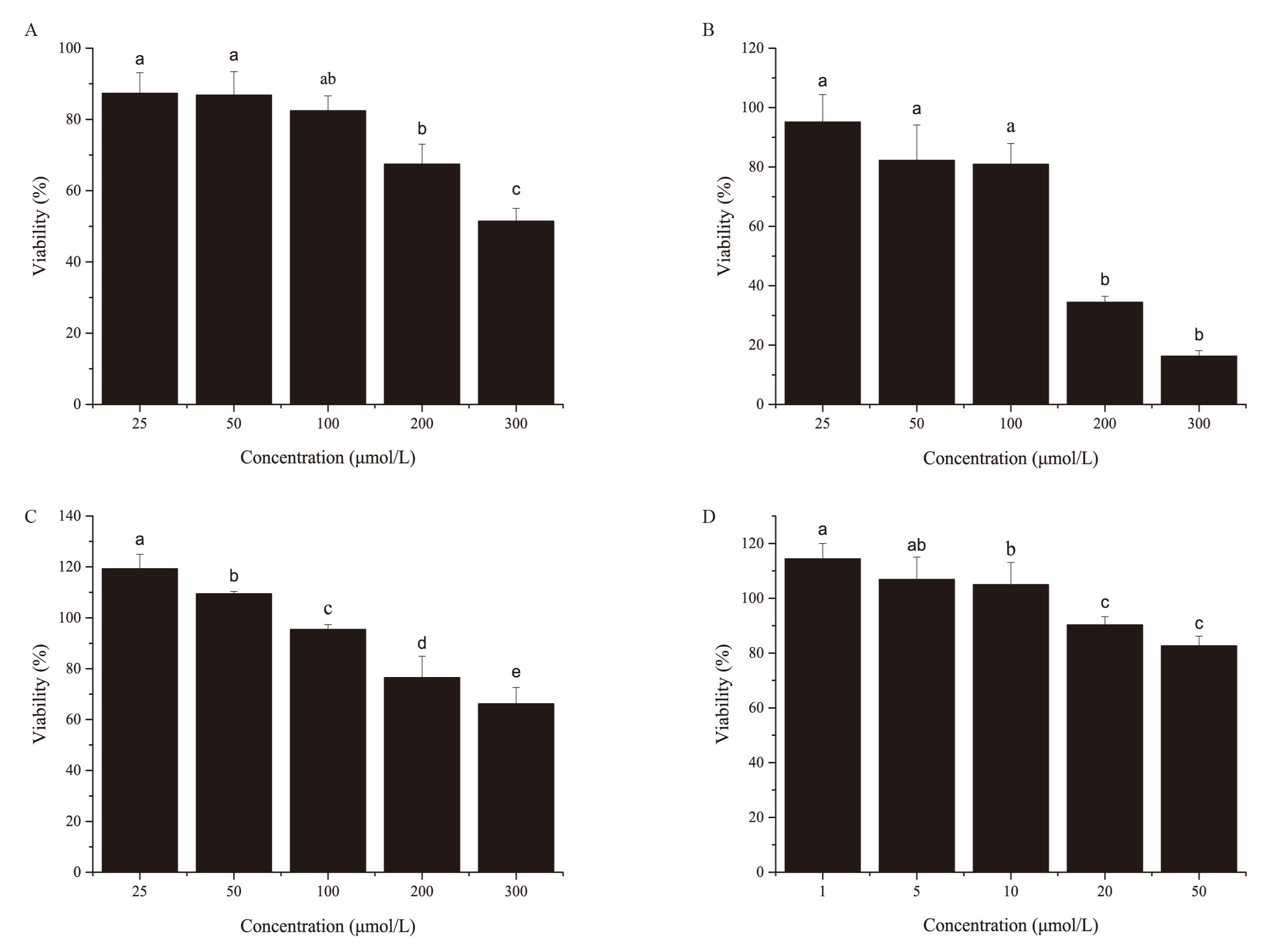


Fig. S2 Effects of four multidrug resistant associated protein inhibitors on the viabilities of Caco-2 cells. (A) verapamil; (B) MK-571; (C) CsA; (D) FTC. Different small letters indicate significant difference between different treatments at p < 0.05.
